# Crystal structure of 3-hy­droxy­methyl-1,2,3,4-tetra­hydro­isoquinolin-1-one

**DOI:** 10.1107/S2056989015012670

**Published:** 2015-07-08

**Authors:** Ignez Caracelli, Camila Lury Hino, Julio Zukerman-Schpector, Francisco Carlos Biaggio, Edward R. T. Tiekink

**Affiliations:** a Departmento de Física, Universidade Federal de São Carlos, 13565-905 São Carlos, SP, Brazil; b Departmento de Química, Universidade Federal de São Carlos, 13565-905 São Carlos, SP, Brazil; c Escola de Engenharia de Lorena - EEL, Universidade São Paulo, SP, Brazil; d Department of Chemistry, University of Malaya, 50603 Kuala Lumpur, Malaysia

**Keywords:** crystal structure, hydrogen bonding, conformation

## Abstract

In the title compound, C_10_H_11_NO_2_, two independent but virtually superimposable mol­ecules, *A* and *B*, comprise the asymmetric unit. The heterocyclic ring in each mol­ecule has a screw-boat conformation, and the methyl­hydroxyl group occupies a position to one side of this ring with N—C—C—O torsion angles of −55.30 (15) (mol­ecule *A*) and −55.94 (16)° (mol­ecule *B*). In the crystal, O—H⋯O and N—H⋯O hydrogen bonding leads to 11-membered {⋯HNCO⋯HO⋯HNC_2_O} heterosynthons, involving three different mol­ecules, which are edge-shared to generate a supra­molecular chain along the *a* axis. Inter­actions of the type C—H⋯O provide additional stability to the chains, and link these into a three-dimensional architecture.

## Related literature   

For background, including medicinal potential, to compounds related to the title compound, see: Biaggio *et al.* (2007 ▸); Grunewald *et al.* (1999 ▸); Zoretic & Soja (1977 ▸). For additional conformational analysis, see: Cremer & Pople (1975 ▸).
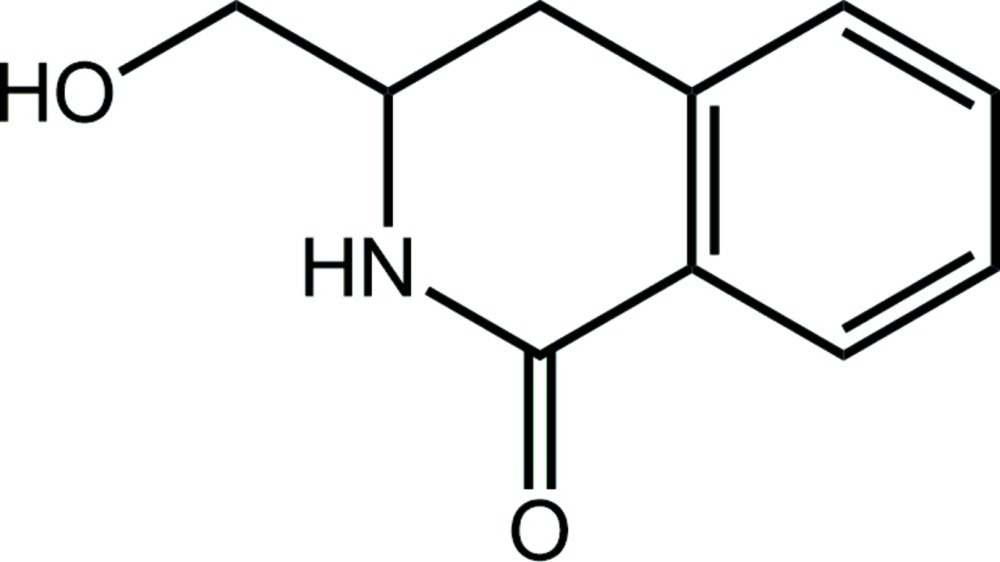


## Experimental   

### Crystal data  

C_10_H_11_NO_2_
*M* *_r_* = 177.20
Orthorhombic, 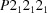

*a* = 6.2846 (1) Å
*b* = 13.8914 (1) Å
*c* = 19.5592 (2) Å
*V* = 1707.56 (3) Å^3^
*Z* = 8
Cu *K*α radiation
μ = 0.79 mm^−1^
*T* = 100 K
0.35 × 0.25 × 0.15 mm

### Data collection  

Agilent SuperNova CCD diffractometer
Absorption correction: multi-scan (*CrysAlis PRO*; Agilent, 2011 ▸) *T*_min_ = 0.882, *T*_max_ = 1.000
6258 measured reflections
3358 independent reflections
3336 reflections with *I* > 2σ(*I*)
*R*_int_ = 0.011

### Refinement  

*R*[*F* ^2^ > 2σ(*F* ^2^)] = 0.026
*wR*(*F* ^2^) = 0.070
*S* = 1.06
3358 reflections
251 parameters
4 restraints
H atoms treated by a mixture of independent and constrained refinement
Δρ_max_ = 0.15 e Å^−3^
Δρ_min_ = −0.29 e Å^−3^
Absolute structure: Flack *x* determined using 1359 quotients [(*I* ^+^)−(*I* ^−^)]/[(*I* ^+^)+(*I* ^−^)] (Parsons *et al.*, 2013 ▸)
Absolute structure parameter: −0.05 (6)

### 

Data collection: *CrysAlis PRO* (Agilent, 2011 ▸); cell refinement: *CrysAlis PRO*; data reduction: *CrysAlis PRO*; program(s) used to solve structure: *SIR2014* (Burla *et al.*, 2015 ▸); program(s) used to refine structure: *SHELXL2014* (Sheldrick, 2015 ▸); molecular graphics: *ORTEP-3 for Windows* (Farrugia, 2012 ▸), *QMOL* (Gans & Shalloway, 2001 ▸) and *DIAMOND* (Brandenburg, 2006 ▸); software used to prepare material for publication: *MarvinSketch* (ChemAxon, 2010 ▸) and *publCIF* (Westrip, 2010 ▸).

## Supplementary Material

Crystal structure: contains datablock(s) I, New_Global_Publ_Block. DOI: 10.1107/S2056989015012670/hg5449sup1.cif

Structure factors: contains datablock(s) I. DOI: 10.1107/S2056989015012670/hg5449Isup2.hkl

Supporting information file. DOI: 10.1107/S2056989015012670/hg5449Isup3.cml

**Fig. 1.**
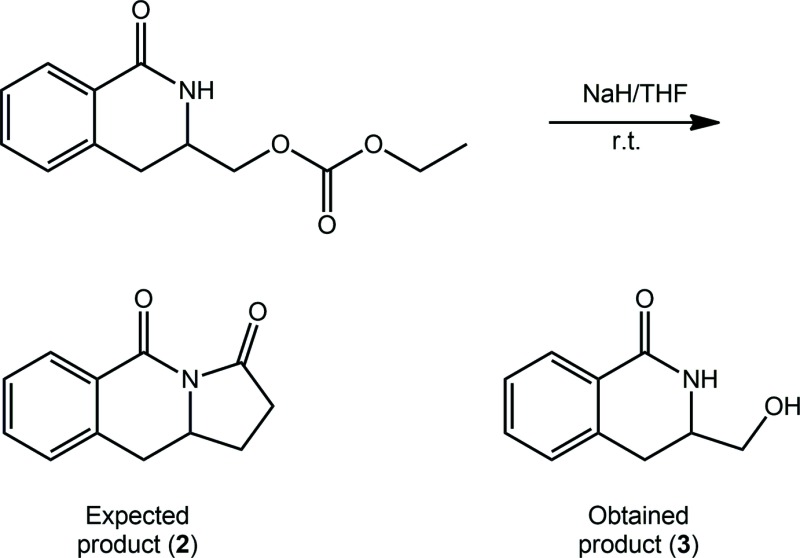
Reaction scheme for the preparation of the title compound.

. DOI: 10.1107/S2056989015012670/hg5449fig1.tif
Reaction scheme for the preparation of the title compound.

**Fig. 2.**
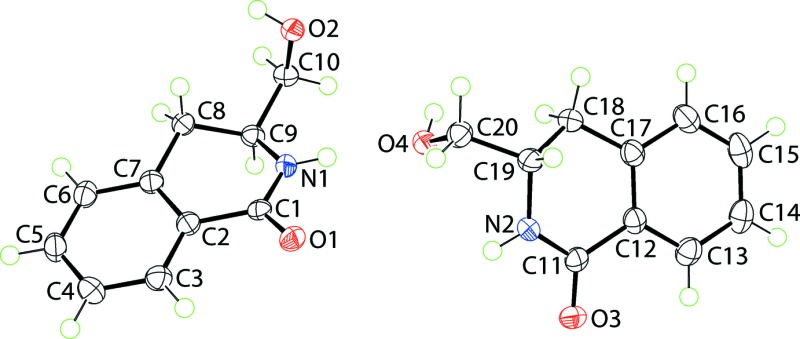
The molecular structures of the two independent molecules in the title compound showing the atom-labelling scheme and displacement ellipsoids at the 70% probability level.

. DOI: 10.1107/S2056989015012670/hg5449fig2.tif
The mol­ecular structures of the two independent mol­ecules in the title compound showing the atom-labelling scheme and displacement ellipsoids at the 70% probability level.

**Fig. 3.**
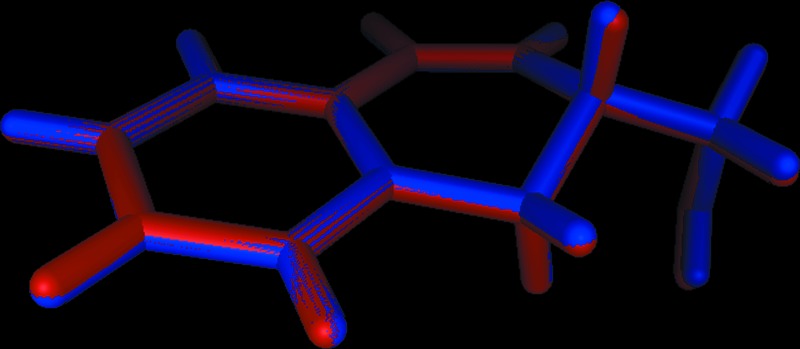
Superimposition of the two independent molecules. Molecule A is shown in blue and B in red. The molecules have been superimposed such that the benzene rings are overlapped.

**Fig. 4.**
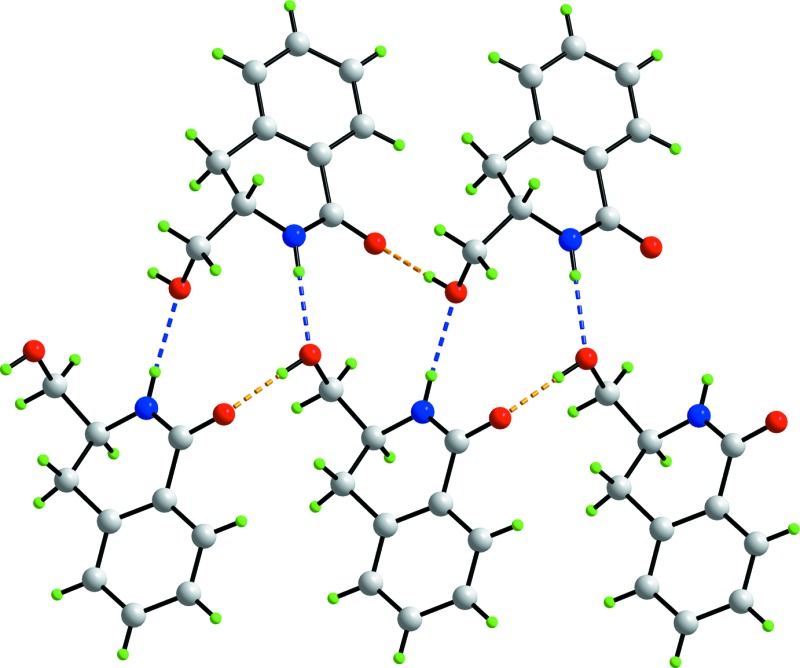
A view of the supramolecular chain sustained by O—H···O and N—H···O hydrogen bonds (orange and blue dashed lines, respectively) and aligned along the a axis in the crystal packing.

**Fig. 5.**
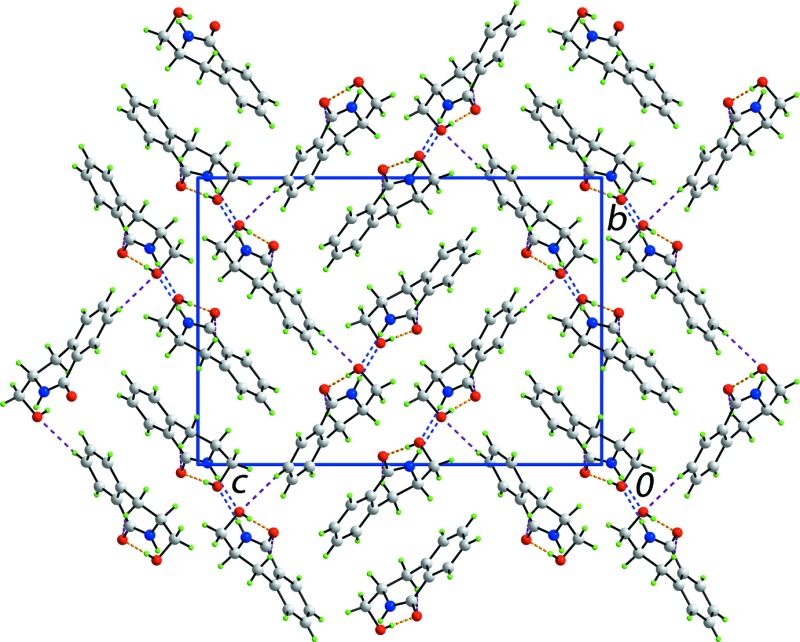
A view in projection down the a axis of the unit-cell contents. The O—H···O, N—H···O and C—H···O interactions are shown as orange, blue and purple dashed lines, respectively.

A B . DOI: 10.1107/S2056989015012670/hg5449fig3.tif
Superimposition of the two independent mol­ecules. Mol­ecule *A* is shown in blue and *B* in red. The mol­ecules have been superimposed such that the benzene rings are overlapped.

a . DOI: 10.1107/S2056989015012670/hg5449fig4.tif
A view of the supra­molecular chain sustained by O—H⋯O and N—H⋯O hydrogen bonds (orange and blue dashed lines, respectively) and aligned along the *a* axis in the crystal packing.

a . DOI: 10.1107/S2056989015012670/hg5449fig5.tif
A view in projection down the *a* axis of the unit-cell contents. The O—H⋯O, N—H⋯O and C—H⋯O inter­actions are shown as orange, blue and purple dashed lines, respectively.

CCDC reference: 1409827

Additional supporting information:  crystallographic information; 3D view; checkCIF report

## Figures and Tables

**Table 1:** Hydrogen-bond geometry (, )

*D*H*A*	*D*H	H*A*	*D* *A*	*D*H*A*
O2H2*O*O1^i^	0.85(1)	1.86(1)	2.7066(14)	176(3)
O4H4*O*O3^i^	0.86(1)	1.83(1)	2.6808(15)	178(3)
N1H1*N*O4	0.86(1)	2.05(1)	2.9141(15)	176(2)
N2H2*N*O2^ii^	0.87(1)	2.00(1)	2.8737(15)	179(2)
C4H4O2^iii^	0.95	2.53	3.2512(18)	132
C8H8*A*O1^i^	0.99	2.48	3.3157(16)	142
C18H18*A*O3^i^	0.99	2.55	3.4007(16)	145

Symmetry codes: (i) 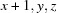; (ii) 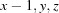; (iii) 
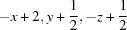
.

## supplementary crystallographic information

### S1. Structural commentary

Referring to Fig. 1, the treatment of la­ctam carbonate (1) with sodium hydride to form compound (2) was unsuccessful and led only to the la­ctam alcohol product (3). Compound (3) might be used as an inter­mediate in organic synthesis. The heterocyclic ring has a screw-boat conformation with puckering parameters (Cremer & Pople, 1975): Puckering Amplitude (Q) = 0.4213 (14) Å, θ = 64.15 (19)° and φ = 271.6 (2)° (molecule *A*), and Q = 0.4050 (15) Å, θ = 64.9 (2)° and φ = 269.3 (2)° (molecule *B*).

### S2. Synthesis and crystallization

A 60% suspension of sodium hydride in mineral oil (22 mg, 1.55 mmol) was suspended in dry tetra­hydro­furan (10 mL; THF) under argon. La­ctam carbonate (1) (250 mg, 1.0 mmol) dissolved in THF (7 mL) was added drop-wise over 5 mins. The resulting reaction mixture was stirred at room temperature for 6 h. The solvent was removed with a rotary evaporator, and the resulting mixture was poured into saturated ammonium hydroxide (10 mL) and extracted with three 20 mL portions of chloro­form. The chloro­form extracts were combined and washed with saturated NaCl solution (20 mL), and dried over anhydrous Mg_2_SO_4_. The solvent was removed and the residue was purified by flash column chromatography (silica gel) with EtOAc as the eluent to yield la­ctam alcohol (3) as a white solid (0.12g, 67%), which was slowly recrystallised from its chloro­form solution (*ca* 7 days). M.pt: 140-142 °C. ^1^H NMR (300 MHz, CDCl_3_): δ 7.20-8.00 (*m*, 4H, ArH), 7.70 (*s*, 1H, NH), 2.0-5.0 (*m*, 5H), 2.02 (*s*, 1H, OH). ^13^C NMR (300 MHz): δ 167.0, 137.9, 132.3, 126.0, 127.0, 128.0, 129.0, 65.0, 53.0, 30.0. IR (KBr, cm^-1^): ν 3684 (OH), 3399, (NH), 1667 (C═O).

### S3. Refinement

Crystal data, data collection and structure refinement details are summarized in Table 1. Carbon-bound H-atoms were placed in calculated positions (C—H = 0.93 to 0.99 Å) and were included in the refinement in the riding model approximation, with *U_iso_*(H) = 1.2–1.5*U_eq_*(C). The O-and N-bound H-atoms were refined with O—H = 0.84±0.01 Å and N—H = 0.86±0.01 Å, and with *U_iso_*(H) = 1.5*U_eq_*(O) or 1.2*U*_eq_(N).

### Crystal data

**Table:**

C_10_H_11_NO_2_	*D*_x_ = 1.379 Mg m^−^^3^
*M**_r_* = 177.20	Cu *K*α radiation, λ = 1.54184 Å
Orthorhombic, *P*2_1_2_1_2_1_	Cell parameters from 5937 reflections
*a* = 6.2846 (1) Å	θ = 3.2–74.2°
*b* = 13.8914 (1) Å	µ = 0.79 mm^−^^1^
*c* = 19.5592 (2) Å	*T* = 100 K
*V* = 1707.56 (3) Å^3^	Prism, colourless
*Z* = 8	0.35 × 0.25 × 0.15 mm
*F*(000) = 752	

### Data collection

**Table:**

Agilent SuperNova CCD diffractometer	3336 reflections with *I* > 2σ(*I*)
Radiation source: SuperNova (Cu) X-ray Source	*R*_int_ = 0.011
ω scans	θ_max_ = 74.3°, θ_min_ = 3.9°
Absorption correction: multi-scan (*CrysAlis PRO*; Agilent, 2011)	*h* = −7→7
*T*_min_ = 0.882, *T*_max_ = 1.000	*k* = −17→17
6258 measured reflections	*l* = −14→24
3358 independent reflections	

### Refinement

**Table:**

Refinement on *F*^2^	Hydrogen site location: mixed
Least-squares matrix: full	H atoms treated by a mixture of independent and constrained refinement
*R*[*F*^2^ > 2σ(*F*^2^)] = 0.026	*w* = 1/[σ^2^(*F*_o_^2^) + (0.0483*P*)^2^ + 0.1971*P*] where *P* = (*F*_o_^2^ + 2*F*_c_^2^)/3
*wR*(*F*^2^) = 0.070	(Δ/σ)_max_ = 0.001
*S* = 1.06	Δρ_max_ = 0.15 e Å^−^^3^
3358 reflections	Δρ_min_ = −0.29 e Å^−^^3^
251 parameters	Absolute structure: Flack *x* determined using 1359 quotients [(*I*^+^)-(*I*^-^)]/[(*I*^+^)+(*I*^-^)] (Parsons *et al.*, 2013)
4 restraints	Absolute structure parameter: −0.05 (6)

### Special details

**Table:**

Geometry. All e.s.d.'s (except the e.s.d. in the dihedral angle between two l.s. planes) are estimated using the full covariance matrix. The cell e.s.d.'s are taken into account individually in the estimation of e.s.d.'s in distances, angles and torsion angles; correlations between e.s.d.'s in cell parameters are only used when they are defined by crystal symmetry. An approximate (isotropic) treatment of cell e.s.d.'s is used for estimating e.s.d.'s involving l.s. planes.

### Fractional atomic coordinates and isotropic or equivalent isotropic displacement parameters (Å^2^)

**Table:**

	*x*	*y*	*z*	*U*_iso_*/*U*_eq_	
O1	0.62669 (16)	0.73762 (7)	0.18705 (5)	0.0180 (2)	
O2	1.32523 (16)	0.66602 (7)	0.10231 (5)	0.0168 (2)	
H2O	1.424 (3)	0.6888 (18)	0.1273 (11)	0.049 (7)*	
N1	0.91512 (19)	0.75379 (8)	0.11906 (6)	0.0150 (2)	
H1N	0.887 (3)	0.6996 (10)	0.0994 (9)	0.021 (5)*	
C1	0.7850 (2)	0.78505 (10)	0.16823 (6)	0.0144 (3)	
C2	0.8387 (2)	0.87911 (10)	0.20099 (7)	0.0156 (3)	
C3	0.6905 (2)	0.92359 (11)	0.24371 (7)	0.0195 (3)	
H3	0.5548	0.8950	0.2507	0.023*	
C4	0.7411 (3)	1.00942 (11)	0.27601 (8)	0.0228 (3)	
H4	0.6397	1.0401	0.3047	0.027*	
C5	0.9411 (3)	1.05064 (11)	0.26623 (8)	0.0212 (3)	
H5	0.9764	1.1091	0.2887	0.025*	
C6	1.0884 (2)	1.00671 (10)	0.22384 (7)	0.0187 (3)	
H6	1.2240	1.0355	0.2172	0.022*	
C7	1.0392 (2)	0.92024 (10)	0.19065 (7)	0.0158 (3)	
C8	1.1995 (2)	0.86671 (10)	0.14844 (7)	0.0169 (3)	
H8A	1.2810	0.8226	0.1784	0.020*	
H8B	1.3008	0.9132	0.1282	0.020*	
C9	1.0952 (2)	0.80877 (10)	0.09148 (7)	0.0153 (3)	
H9	1.0405	0.8544	0.0561	0.018*	
C10	1.2538 (2)	0.74041 (10)	0.05779 (7)	0.0163 (3)	
H10A	1.1863	0.7108	0.0172	0.020*	
H10B	1.3783	0.7779	0.0419	0.020*	
O3	0.10908 (16)	0.52866 (7)	−0.04188 (5)	0.0200 (2)	
O4	0.80454 (17)	0.57641 (7)	0.04790 (5)	0.0183 (2)	
H4O	0.904 (3)	0.5612 (18)	0.0200 (11)	0.050 (7)*	
N2	0.3944 (2)	0.49397 (8)	0.02382 (6)	0.0165 (2)	
H2N	0.373 (4)	0.5458 (11)	0.0481 (9)	0.028 (5)*	
C11	0.2649 (2)	0.47641 (10)	−0.02887 (7)	0.0160 (3)	
C12	0.3141 (2)	0.39062 (10)	−0.07222 (7)	0.0174 (3)	
C13	0.1653 (2)	0.35932 (11)	−0.12001 (7)	0.0217 (3)	
H13	0.0327	0.3916	−0.1242	0.026*	
C14	0.2107 (3)	0.28085 (12)	−0.16165 (7)	0.0241 (3)	
H14	0.1092	0.2591	−0.1941	0.029*	
C15	0.4058 (3)	0.23446 (11)	−0.15537 (8)	0.0249 (3)	
H15	0.4370	0.1805	−0.1835	0.030*	
C16	0.5553 (3)	0.26632 (11)	−0.10831 (8)	0.0230 (3)	
H16	0.6883	0.2342	−0.1047	0.028*	
C17	0.5124 (2)	0.34507 (10)	−0.06620 (7)	0.0188 (3)	
C18	0.6750 (2)	0.38637 (10)	−0.01815 (8)	0.0208 (3)	
H18A	0.7610	0.4350	−0.0428	0.025*	
H18B	0.7720	0.3344	−0.0031	0.025*	
C19	0.5744 (2)	0.43317 (10)	0.04445 (7)	0.0179 (3)	
H19	0.5197	0.3811	0.0751	0.022*	
C20	0.7337 (2)	0.49417 (10)	0.08474 (7)	0.0194 (3)	
H20A	0.6665	0.5155	0.1279	0.023*	
H20B	0.8583	0.4539	0.0966	0.023*	

### Atomic displacement parameters (Å^2^)

**Table:**

	*U*^11^	*U*^22^	*U*^33^	*U*^12^	*U*^13^	*U*^23^
O1	0.0137 (5)	0.0198 (5)	0.0204 (5)	−0.0022 (4)	0.0008 (4)	−0.0006 (4)
O2	0.0153 (5)	0.0148 (5)	0.0203 (5)	0.0004 (4)	−0.0012 (4)	−0.0004 (4)
N1	0.0141 (5)	0.0138 (5)	0.0171 (5)	−0.0010 (4)	−0.0001 (4)	−0.0022 (4)
C1	0.0131 (6)	0.0157 (6)	0.0145 (6)	0.0020 (5)	−0.0029 (5)	0.0016 (5)
C2	0.0162 (7)	0.0161 (6)	0.0146 (6)	0.0009 (5)	−0.0017 (5)	0.0005 (5)
C3	0.0163 (7)	0.0229 (7)	0.0193 (6)	0.0007 (6)	0.0009 (6)	−0.0021 (5)
C4	0.0222 (7)	0.0242 (7)	0.0219 (7)	0.0035 (6)	0.0021 (6)	−0.0061 (6)
C5	0.0247 (8)	0.0180 (7)	0.0210 (7)	−0.0005 (6)	−0.0045 (6)	−0.0050 (5)
C6	0.0193 (7)	0.0172 (6)	0.0196 (7)	−0.0026 (6)	−0.0027 (5)	−0.0004 (5)
C7	0.0170 (7)	0.0154 (6)	0.0152 (6)	0.0011 (5)	−0.0021 (5)	0.0015 (5)
C8	0.0131 (6)	0.0162 (6)	0.0216 (7)	−0.0017 (5)	0.0005 (6)	−0.0017 (5)
C9	0.0137 (6)	0.0153 (6)	0.0167 (6)	0.0003 (5)	0.0015 (5)	0.0009 (5)
C10	0.0158 (6)	0.0179 (6)	0.0152 (6)	0.0013 (5)	0.0013 (5)	0.0013 (5)
O3	0.0151 (5)	0.0225 (5)	0.0225 (5)	0.0022 (4)	−0.0001 (4)	−0.0020 (4)
O4	0.0162 (5)	0.0161 (5)	0.0227 (5)	−0.0004 (4)	0.0016 (4)	−0.0021 (4)
N2	0.0163 (6)	0.0150 (5)	0.0183 (5)	0.0013 (5)	0.0003 (5)	−0.0032 (4)
C11	0.0143 (6)	0.0167 (6)	0.0172 (6)	−0.0033 (5)	0.0034 (5)	0.0004 (5)
C12	0.0179 (7)	0.0169 (6)	0.0174 (6)	−0.0030 (5)	0.0034 (5)	−0.0017 (5)
C13	0.0191 (7)	0.0253 (7)	0.0206 (7)	−0.0040 (6)	0.0026 (6)	−0.0014 (6)
C14	0.0275 (8)	0.0260 (7)	0.0187 (7)	−0.0089 (6)	0.0018 (6)	−0.0036 (6)
C15	0.0348 (9)	0.0187 (6)	0.0212 (7)	−0.0057 (7)	0.0083 (6)	−0.0046 (6)
C16	0.0250 (7)	0.0173 (7)	0.0267 (7)	0.0007 (6)	0.0057 (6)	−0.0022 (6)
C17	0.0195 (7)	0.0159 (6)	0.0210 (6)	−0.0020 (5)	0.0034 (5)	−0.0006 (5)
C18	0.0156 (7)	0.0180 (6)	0.0288 (7)	0.0018 (5)	−0.0001 (6)	−0.0040 (6)
C19	0.0169 (6)	0.0153 (6)	0.0215 (7)	0.0011 (5)	−0.0018 (5)	0.0019 (5)
C20	0.0185 (7)	0.0200 (6)	0.0198 (6)	−0.0005 (6)	−0.0026 (5)	0.0005 (5)

### Geometric parameters (Å, º)

**Table:**

O1—C1	1.2486 (17)	O3—C11	1.2454 (18)
O2—C10	1.4239 (16)	O4—C20	1.4220 (17)
O2—H2O	0.849 (13)	O4—H4O	0.855 (13)
N1—C1	1.3351 (18)	N2—C11	1.3356 (18)
N1—C9	1.4680 (17)	N2—C19	1.4682 (18)
N1—H1N	0.863 (12)	N2—H2N	0.872 (12)
C1—C2	1.4939 (18)	C11—C12	1.4949 (18)
C2—C3	1.396 (2)	C12—C13	1.392 (2)
C2—C7	1.398 (2)	C12—C17	1.402 (2)
C3—C4	1.386 (2)	C13—C14	1.390 (2)
C3—H3	0.9500	C13—H13	0.9500
C4—C5	1.395 (2)	C14—C15	1.391 (2)
C4—H4	0.9500	C14—H14	0.9500
C5—C6	1.385 (2)	C15—C16	1.388 (2)
C5—H5	0.9500	C15—H15	0.9500
C6—C7	1.400 (2)	C16—C17	1.396 (2)
C6—H6	0.9500	C16—H16	0.9500
C7—C8	1.4997 (19)	C17—C18	1.502 (2)
C8—C9	1.5227 (18)	C18—C19	1.524 (2)
C8—H8A	0.9900	C18—H18A	0.9900
C8—H8B	0.9900	C18—H18B	0.9900
C9—C10	1.5263 (19)	C19—C20	1.5303 (19)
C9—H9	1.0000	C19—H19	1.0000
C10—H10A	0.9900	C20—H20A	0.9900
C10—H10B	0.9900	C20—H20B	0.9900
			
C10—O2—H2O	108.1 (18)	C20—O4—H4O	110.7 (17)
C1—N1—C9	124.60 (12)	C11—N2—C19	125.20 (12)
C1—N1—H1N	118.7 (14)	C11—N2—H2N	118.6 (14)
C9—N1—H1N	116.5 (14)	C19—N2—H2N	116.2 (14)
O1—C1—N1	121.92 (13)	O3—C11—N2	122.02 (13)
O1—C1—C2	121.01 (12)	O3—C11—C12	120.76 (12)
N1—C1—C2	117.06 (12)	N2—C11—C12	117.21 (12)
C3—C2—C7	120.47 (13)	C13—C12—C17	120.82 (13)
C3—C2—C1	119.55 (13)	C13—C12—C11	119.41 (13)
C7—C2—C1	119.94 (12)	C17—C12—C11	119.73 (13)
C4—C3—C2	120.03 (14)	C14—C13—C12	120.04 (15)
C4—C3—H3	120.0	C14—C13—H13	120.0
C2—C3—H3	120.0	C12—C13—H13	120.0
C3—C4—C5	119.83 (14)	C13—C14—C15	119.50 (14)
C3—C4—H4	120.1	C13—C14—H14	120.3
C5—C4—H4	120.1	C15—C14—H14	120.3
C6—C5—C4	120.26 (13)	C16—C15—C14	120.51 (14)
C6—C5—H5	119.9	C16—C15—H15	119.7
C4—C5—H5	119.9	C14—C15—H15	119.7
C5—C6—C7	120.54 (14)	C15—C16—C17	120.70 (15)
C5—C6—H6	119.7	C15—C16—H16	119.7
C7—C6—H6	119.7	C17—C16—H16	119.7
C2—C7—C6	118.86 (13)	C16—C17—C12	118.42 (14)
C2—C7—C8	118.83 (12)	C16—C17—C18	122.47 (14)
C6—C7—C8	122.17 (13)	C12—C17—C18	119.00 (12)
C7—C8—C9	112.08 (12)	C17—C18—C19	112.53 (12)
C7—C8—H8A	109.2	C17—C18—H18A	109.1
C9—C8—H8A	109.2	C19—C18—H18A	109.1
C7—C8—H8B	109.2	C17—C18—H18B	109.1
C9—C8—H8B	109.2	C19—C18—H18B	109.1
H8A—C8—H8B	107.9	H18A—C18—H18B	107.8
N1—C9—C8	109.74 (11)	N2—C19—C18	110.14 (11)
N1—C9—C10	109.78 (11)	N2—C19—C20	109.10 (11)
C8—C9—C10	111.33 (11)	C18—C19—C20	112.23 (12)
N1—C9—H9	108.6	N2—C19—H19	108.4
C8—C9—H9	108.6	C18—C19—H19	108.4
C10—C9—H9	108.6	C20—C19—H19	108.4
O2—C10—C9	113.17 (11)	O4—C20—C19	112.88 (11)
O2—C10—H10A	108.9	O4—C20—H20A	109.0
C9—C10—H10A	108.9	C19—C20—H20A	109.0
O2—C10—H10B	108.9	O4—C20—H20B	109.0
C9—C10—H10B	108.9	C19—C20—H20B	109.0
H10A—C10—H10B	107.8	H20A—C20—H20B	107.8
			
C9—N1—C1—O1	175.75 (12)	C19—N2—C11—O3	176.74 (13)
C9—N1—C1—C2	−5.26 (19)	C19—N2—C11—C12	−3.2 (2)
O1—C1—C2—C3	−12.24 (19)	O3—C11—C12—C13	−11.1 (2)
N1—C1—C2—C3	168.76 (13)	N2—C11—C12—C13	168.84 (13)
O1—C1—C2—C7	165.51 (12)	O3—C11—C12—C17	166.40 (13)
N1—C1—C2—C7	−13.49 (18)	N2—C11—C12—C17	−13.68 (19)
C7—C2—C3—C4	0.3 (2)	C17—C12—C13—C14	1.2 (2)
C1—C2—C3—C4	178.05 (13)	C11—C12—C13—C14	178.68 (13)
C2—C3—C4—C5	−0.6 (2)	C12—C13—C14—C15	−0.4 (2)
C3—C4—C5—C6	0.7 (2)	C13—C14—C15—C16	−0.4 (2)
C4—C5—C6—C7	−0.4 (2)	C14—C15—C16—C17	0.3 (2)
C3—C2—C7—C6	0.0 (2)	C15—C16—C17—C12	0.5 (2)
C1—C2—C7—C6	−177.76 (12)	C15—C16—C17—C18	−175.52 (14)
C3—C2—C7—C8	175.65 (13)	C13—C12—C17—C16	−1.3 (2)
C1—C2—C7—C8	−2.08 (18)	C11—C12—C17—C16	−178.75 (13)
C5—C6—C7—C2	0.1 (2)	C13—C12—C17—C18	174.88 (13)
C5—C6—C7—C8	−175.46 (13)	C11—C12—C17—C18	−2.57 (19)
C2—C7—C8—C9	33.21 (17)	C16—C17—C18—C19	−151.24 (14)
C6—C7—C8—C9	−151.25 (13)	C12—C17—C18—C19	32.74 (18)
C1—N1—C9—C8	35.96 (18)	C11—N2—C19—C18	32.98 (18)
C1—N1—C9—C10	158.62 (12)	C11—N2—C19—C20	156.58 (12)
C7—C8—C9—N1	−47.52 (15)	C17—C18—C19—N2	−45.45 (16)
C7—C8—C9—C10	−169.26 (11)	C17—C18—C19—C20	−167.21 (12)
N1—C9—C10—O2	−55.30 (15)	N2—C19—C20—O4	−55.94 (16)
C8—C9—C10—O2	66.42 (15)	C18—C19—C20—O4	66.41 (16)

### Hydrogen-bond geometry (Å, º)

**Table:**

*D*—H···*A*	*D*—H	H···*A*	*D*···*A*	*D*—H···*A*
O2—H2*O*···O1^i^	0.85 (1)	1.86 (1)	2.7066 (14)	176 (3)
O4—H4*O*···O3^i^	0.86 (1)	1.83 (1)	2.6808 (15)	178 (3)
N1—H1*N*···O4	0.86 (1)	2.05 (1)	2.9141 (15)	176 (2)
N2—H2*N*···O2^ii^	0.87 (1)	2.00 (1)	2.8737 (15)	179 (2)
C4—H4···O2^iii^	0.95	2.53	3.2512 (18)	132
C8—H8*A*···O1^i^	0.99	2.48	3.3157 (16)	142
C18—H18*A*···O3^i^	0.99	2.55	3.4007 (16)	145

Symmetry codes: (i) *x*+1, *y*, *z*; (ii) *x*−1, *y*, *z*; (iii) −*x*+2, *y*+1/2, −*z*+1/2.
