# Supplementary figures and images for: Crystal structure of 3-hy­droxy­methyl-1,2,3,4-tetra­hydro­isoquinolin-1-one
Source: Acta Crystallogr E Crystallogr Commun. 2015 Jul 8;71(Pt 8):o558–9. doi: 10.1107/S2056989015012670 (PMC4571395; doi:10.1107/S2056989015012670)

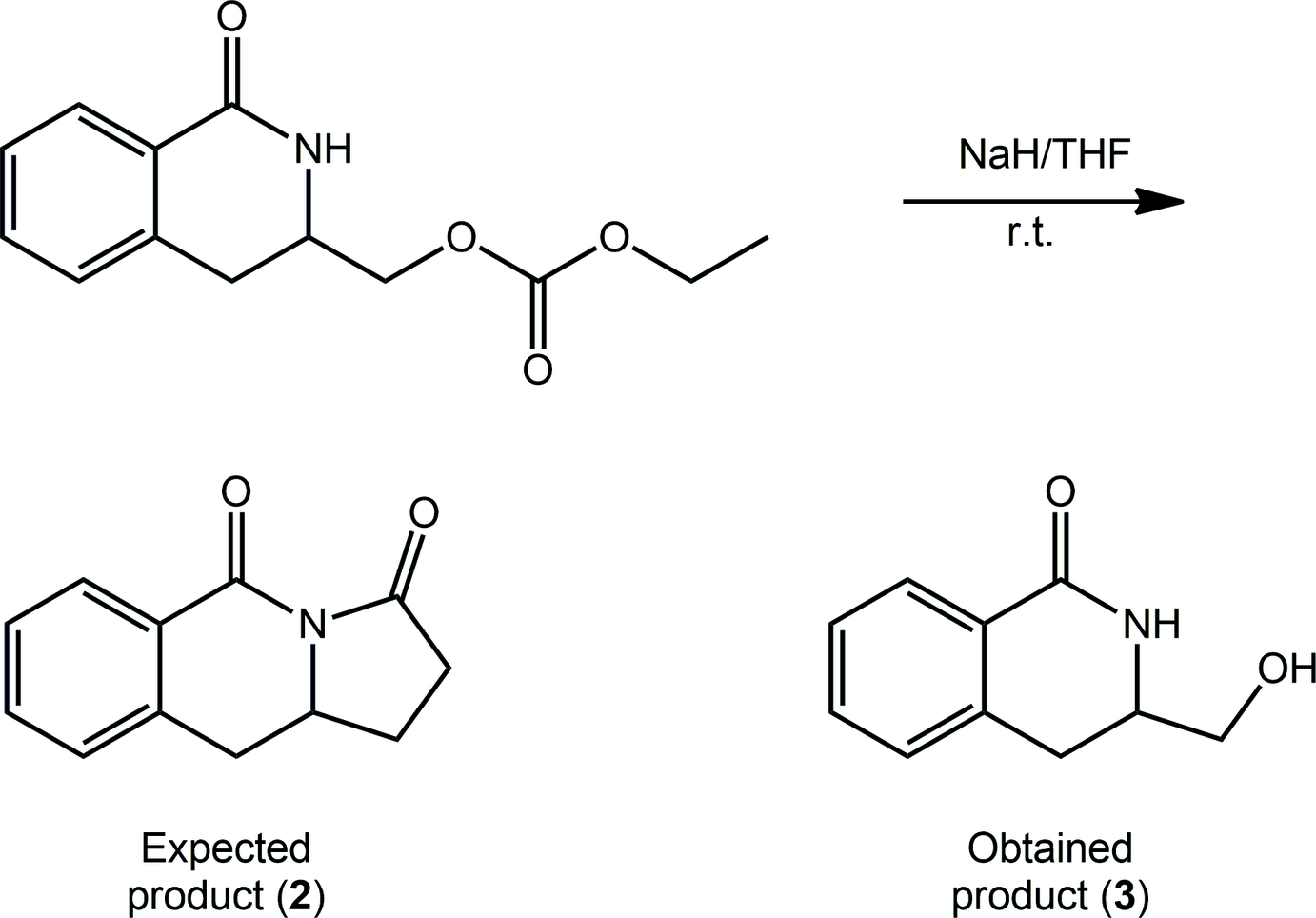

Supplement: Supplementary file 4 [file e-71-0o558-fig1.tif]

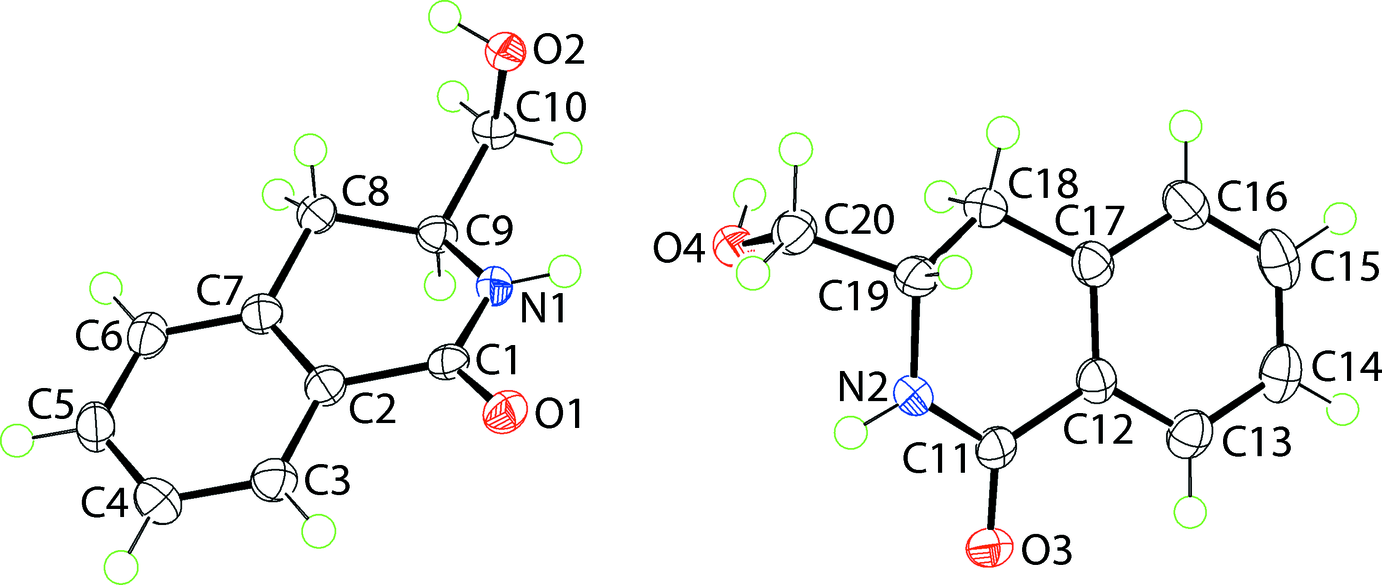

Supplement: Supplementary file 5 [file e-71-0o558-fig2.tif]

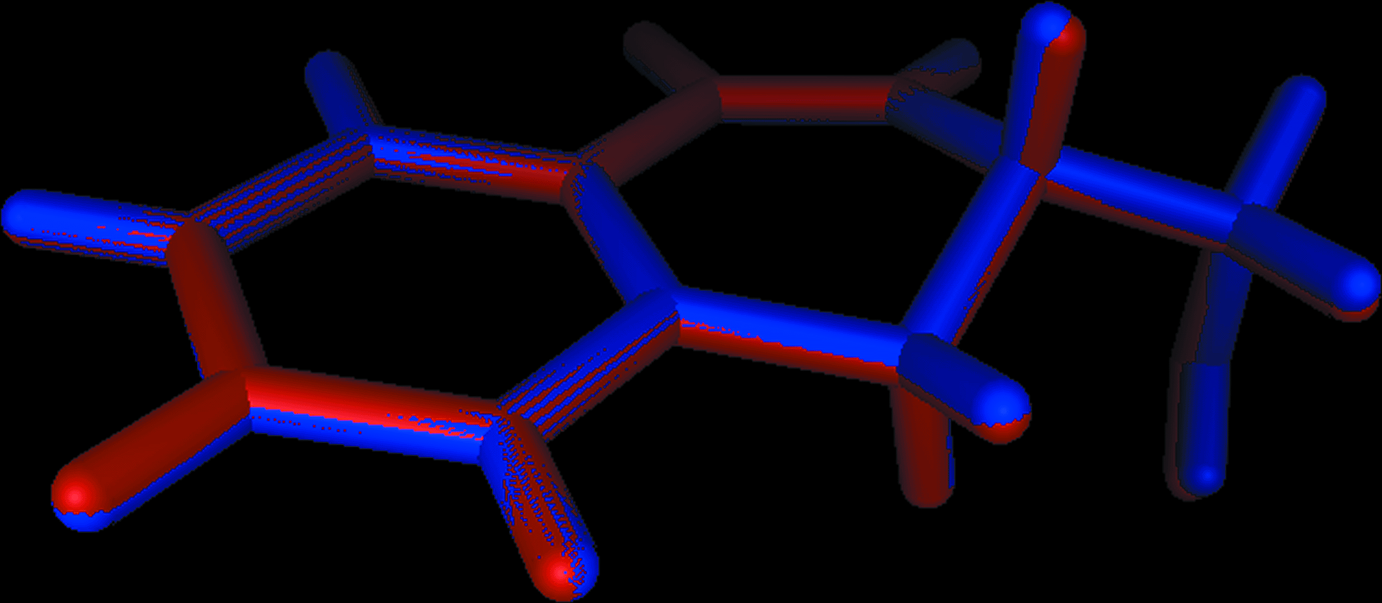

Supplement: Supplementary file 6 [file e-71-0o558-fig3.tif]

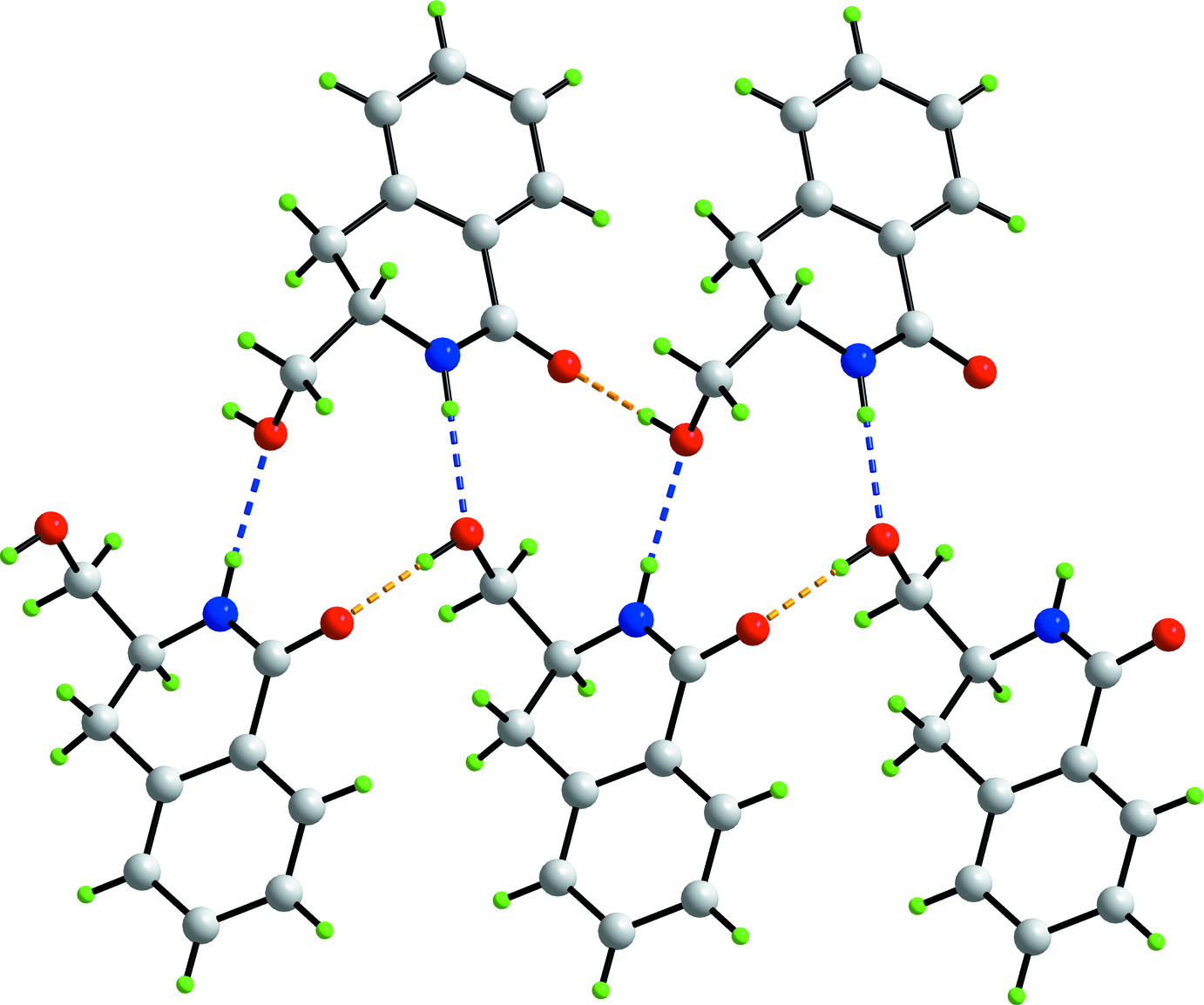

Supplement: Supplementary file 7 [file e-71-0o558-fig4.tif]

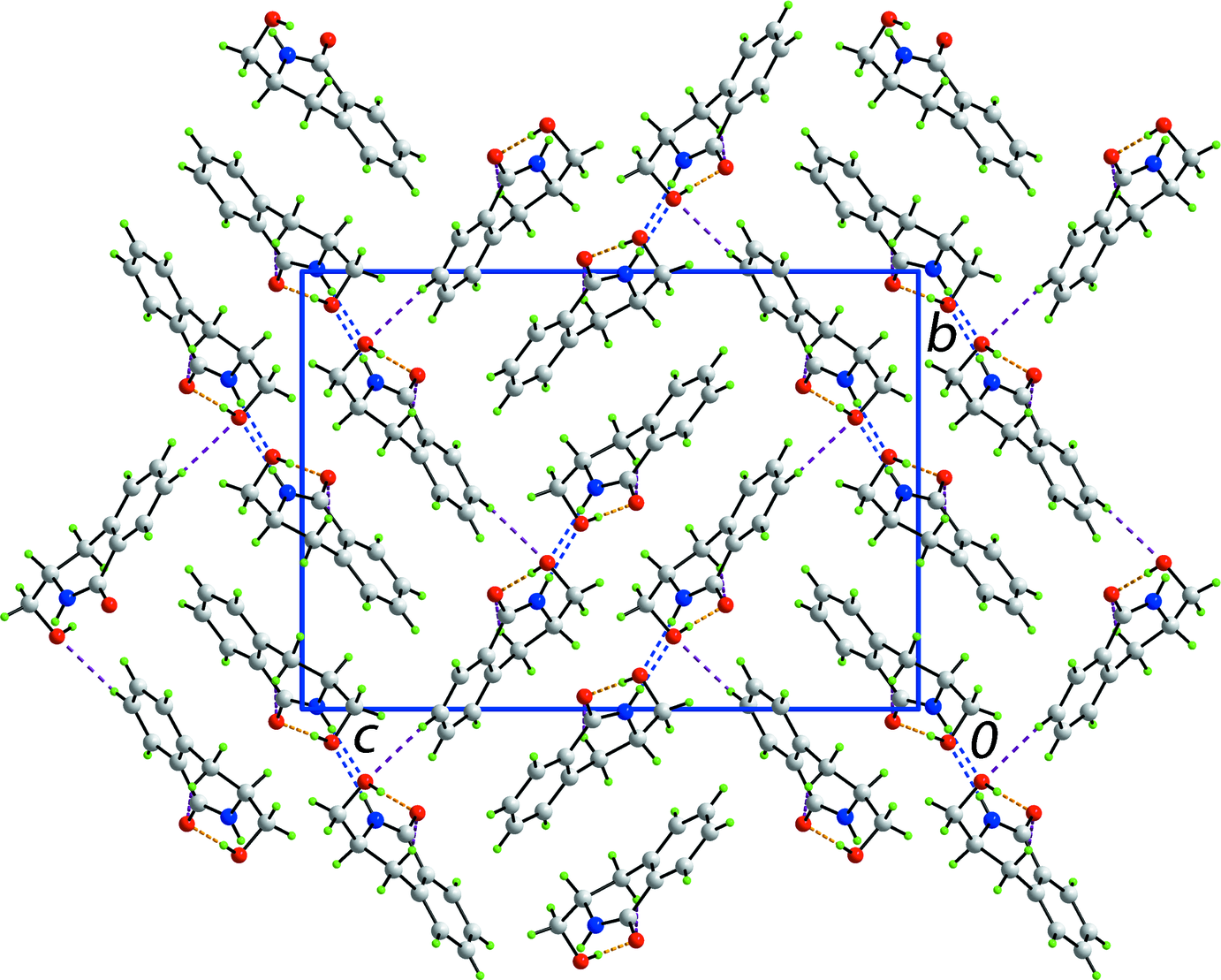

Supplement: Supplementary file 8 [file e-71-0o558-fig5.tif]
